# Characteristics and Outcomes of Younger Adults with Embolic Stroke of Undetermined Source (ESUS): A Retrospective Study

**DOI:** 10.1155/2019/4360787

**Published:** 2019-12-03

**Authors:** Ali M. Al Khathaami, Bayan Al Bdah, Abdulmjeed Alnosair, Abdulkarim Alturki, Rayan Alrebdi, Shorug Alwayili, Sulaiman Alhamzah, Fahad A. M. AlKhathaami, Nasser Alotaibi

**Affiliations:** ^1^King Abdulaziz Medical City, National Guard Health Affairs, Riyadh, Saudi Arabia; ^2^College of Medicine, King Saud Bin Abdul Aziz University for Health Sciences, Riyadh, Saudi Arabia; ^3^King Abdullah International Medical Research Center, Saudi Arabia

## Abstract

**Introduction:**

Embolic stroke of undetermined source (ESUS) in younger adults may have different risk factors compared with ESUS in elderly, and the approach to ESUS in young adults may require new therapies. We aimed to investigate the characteristics and outcomes in younger adults with ESUS at a single centre in Saudi Arabia.

**Patients and Methods:**

A retrospective study was conducted using the medical records of younger adults with ESUS according to the criteria of the Cryptogenic Stroke/ESUS International Working Group. Younger adults (aged ≤50 years) with ESUS were compared with older patients, on admission and discharge from hospital, using the modified Rankin scale (mRS) and the National Institute of Health Stroke Scale (NIHSS).

**Results:**

Among 147 patients with ESUS, 39 (26.5%) were younger adults. Younger adults compared with older adults with ESUS had fewer vascular risk factors, including lower rates of hypertension (43.6% vs. 70.3%; *P* = 0.004), diabetes (35.9% vs. 57.4%; *P* = 0.03), and dyslipidaemia (12.8% vs. 28.7%; *P* = 0.05). There was no significant difference in poor outcome at discharge (defined as mRS > 2), which was observed in 17.9% of younger adults and 28.7% of older adults. Further, there were no significant differences in stroke severity at discharge (NIHSS score ≤5) or median length of stay.

**Discussion:**

Although the outcomes of ESUS do not differ between younger and older patients, younger patients have fewer identified risk factors.

**Conclusion:**

This study showed that 26.5% of patients with ESUS were aged ≤50  years. Although younger adults with ESUS had fewer risk factors, there were no significant differences in neurologic disability or mortality at discharge, stroke severity, or median length of stay.

## 1. Introduction

Stroke in younger adults aged ≤50 years is a common condition in clinical practice, with an estimated incidence from 5.8 to 11.4 per 100,000 [1–6]. This subgroup requires a different approach to diagnosis and management, as their cardiovascular risk factors, aetiology, and prognosis differ from those of older patients with ischaemic stroke [7, 8]. The recognition of these age-related differences is essential for optimal investigation, treatment, and prevention.

Embolic stroke of undetermined source (ESUS) is a newly described type of ischaemic stroke where thrombo-embolism is the most likely cause. ESUS is defined as a non-lacunar brain infarct (detected by computed tomography or magnetic resonance imaging) in the absence of extracranial or intracranial atherosclerosis causing ≥50% luminal stenosis in arteries supplying the ischaemic area, a major-risk cardioembolic source, and any other specific cause of stroke [9]. The prevalence of ESUS ranges from 7% to 42% among patients with ischaemic stroke [10]. In general, patients with ESUS are younger, have fewer vascular comorbidities, and may have more favourable outcomes than patients with other types of stroke [11–14]. Little is known about ESUS in younger adults compared with older patients.

This study aimed to investigate the characteristics and clinical outcomes of younger adults with ESUS at a single centre in Saudi Arabia.

## 2. Patients and Methods

### 2.1. Study Design

A retrospective review was undertaken of patients with ischaemic stroke who were admitted to the Acute Stroke Unit at King Abdulaziz Medical City, Riyadh (KAMC-R), Saudi Arabia, from February 2016 to July 2018. KAMC-R has more than a thousand beds and is a Joint Commission-accredited academic and tertiary centre, treating an average of 500 patients with stroke each year. The stroke unit is divided into two levels of care: the Hyperacute Stroke Unit with cardiopulmonary monitoring for the first 72 hours after admission and the Acute Stroke Unit. The unit is run by specialised stroke neurologists and a multidisciplinary team. Clinical care pathways and best practices govern patient care.

All patients with stroke were admitted to the hyperacute stroke unit under cardiopulmonary monitoring for 72 hours and subsequently shifted to the acute stroke unit. All ischaemic stroke patients had routine laboratory investigations and transthoracic echocardiography (TTE), brain computed tomography (CT), and computed tomography angiography (CTA) of the carotid arteries and the circle of Willis. If the use of CTA is contra-indicated, doppler ultrasound or magnetic resonance (MR) angiography was performed. Magnetic resonance imaging (MRI) of the brain was performed if CT scan of the brain did not show the infarct pattern, when cerebral vasculitis is suspected, when the ischemic stroke classification is uncertain or when patient is aged <50 years. Patients aged <50 years with no apparent cause of stroke undergo further testing for vasculitis and hypercoagulability. Erythrocyte sedimentation rate (ESR) and an elevated C-reactive protein, antinuclear antibody, rheumatoid factor, complements, cryoglobulins, anti-neutrophil cytoplasmic antibodies, hepatitis B and C serology, lupus anticoagulant, anticardiolipin antibodies, Beta-2 glycoprotein antibodies, factor V Leiden mutation, prothrombin gene mutation, protein C deficiency, protein S deficiency, anti-thrombin deficiency, and homocysteine were performed. Further, all patients less than 50 years with no apparent cause underwent transoesophageal echocardiography (TEE). If cardioembolic stroke is still suspected, patient may have outpatient multiple Holter monitors and/or prolonged cardiac rhythm monitoring through insertable cardiac monitor device. The stroke unit admission criteria comprised having confirmed or probable stroke or transient ischaemic attack (TIA) at initial presentation, being aged <80 years, having a modified Rankin scale (mRS) score ≤2 before the stroke, and having no history of dementia or terminal illness. Those older than 80 years with poor functional status (mRS > 2) or had dementia or terminal illness were admitted under general medicine outside the stroke unit and they were excluded from this study.

### 2.2. Data Collection and Identification of Cases of Embolic Stroke of Undetermined Source (ESUS)

**Figure 1:**
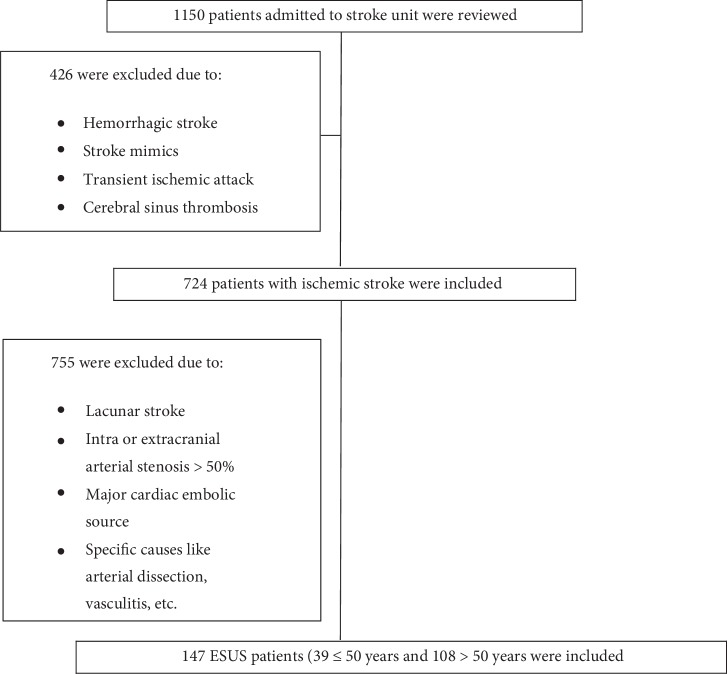
Flow chart of patient inclusion/exclusion. ∗Major cardiac source includes atrial fibrillation (permanent or paroxysmal), sustained atrial flutter, intracardiac thrombus, prosthetic cardiac valve, cardiac tumors, mitral stenosis, recent (<4 weeks) myocardial infarction, left ventricular ejection fraction <30%, valvular vegetations, or infective endocarditis.

Data on stroke subtype, length of hospital stay, mRS score at hospital admission and discharge, National Institute of Health Stroke Scale (NIHSS) score at admission and discharge, patient demographics, vascular risk factors, comorbidities, echocardiogram, vascular images, and laboratory findings were retrospectively obtained from electronic health records. After exclusion of haemorrhagic stroke, transient ischemic attacks, stroke mimics and cerebral sinus thrombosis, we identified 724 ischaemic stroke patients. Eighty one percent had brain MRI. The remaining 19% had brain CT that showed unequivocal embolic nature of stroke. All patient had vascular images, TTE and routine laboratory investigations. The criteria proposed by the Cryptogenic Stroke/ESUS International Working Group were applied, which defines ESUS as a nonlacunar brain infarct (detected by computed tomography or magnetic resonance imaging) in in the absence of extracranial or intracranial atherosclerosis causing ≥50% stenosis in arteries supplying the ischaemic area, major-risk cardioembolic source, and any other specific cause of stroke (Figure 1) [9]. The research team underwent training to use the ESUS criteria. In cases of dispute, the team members discussed the cases individually and disagreement was resolved by consensus.

### 2.3. Data Analysis

Patients who met the diagnostic criteria for ESUS were included in the analysis. The cohort of younger patients aged ≤50 years was compared with the older patients aged >50 years. Poor outcome at discharge (defined as mRS score >2), length of hospital stay, and NIHSS score at discharge were compared between the two groups. Data were presented as the mean ± standard deviation (SD) for continuous variables and as percentages for categorical variables. Student's *t*-test and the chi-square (*χ*^2^) test were used to compare the means and proportions, respectively. Clinical outcomes at discharge (poor outcome and NIHSS score ≤5) were compared between the two groups using multivariate logistic regression analyses, including both a nonadjusted analysis and an analysis that adjusted for age, gender, comorbidities, and stroke severity (NIHSS score at admission ≤5 vs. >5). To compare the length of hospital, stay between the two groups, multivariate quantile regression analyses were conducted, including both a nonadjusted analysis and an analysis that adjusted for age, gender, comorbidities, stroke severity, and stroke classification. Data were analysed using Stata version 15 (Stata Corporation, College Station, TX, USA). A *P*-value <0.05 was considered to be statistically significant.

### 2.4. Ethical Aspects

The study was approved by local IRB.

## 3. Results

### 3.1. Patient Characteristics

Between February 2016 and July 2018, 147 patients were admitted to the Acute Stroke Unit with embolic stroke of undetermined source (ESUS). There were 39 (26.5%) patients who were younger adults (≤50 years). The baseline characteristics of the two groups (≤50 vs. >50 years) are shown in Table 1. Younger adults with ESUS had fewer vascular risk factors, with lower rates of arterial hypertension (43.6% vs. 70.3%; *P* = 0.004), diabetes mellitus (35.9% vs. 57.4%; *P* = 0.03), and dyslipidaemia (12.8% vs. 28.7%; *P* = 0.05). There was no significant difference in the median NIHSS score at admission between the two groups (3 vs. 5; *P* = 0.8).

### 3.2. Patient Outcomes

Patient outcome data are summarised in Table 2. In the nonadjusted logistic regression analysis, there was no significant difference between the younger and older patients with ESUS in terms of poor outcome (neurological disability or mortality defined as mRS score >2 at discharge, 17.9% vs. 28.7%; OR, 1.8; 95% CI, 0.7–4.6; *P* = 0.2). The proportion of patients with no or mild neurological deficit at discharge (NIHSS score ≤5) was similar in the two groups (84.6% vs. 74.8%; OR, 1.9; 95% CI, 0.7–4.9; *P* = 0.2). The median length of hospital stay was not significantly different between the two groups (4.0 vs. 4.0 days; *P* = 0.3). After adjustment for gender, vascular risk factors, stroke severity, and treatment with tissue plasminogen activator or thrombectomy in the multivariate regression analyses, there were no significant differences between the two groups in terms of poor outcome (neurological disability or mortality at discharge), no or mild neurological deficit at discharge (NIHSS score ≤5), or median length of hospital stay.

## 4. Discussion

Worldwide, ischaemic stroke affects nearly two million younger adults each year [15, 16]. In contrast to stroke in the older population, the incidence of stroke in younger people (aged ≤50 years) is increasing [16]. It is estimated that one in ten strokes involve younger adults, resulting in a significant socioeconomic impact [16]. This subgroup of younger patients with ischaemic stroke is typically under-represented in clinical trials, and it can be challenging to extrapolate evidence from the older stroke population to the younger stroke population.

Since the first description of embolic stroke of undetermined source (ESUS) in 2014, there has been increasing interest in ESUS-specific treatment approaches, as anticoagulants may reduce recurrent cases of ESUS more effectively than antiplatelet drugs [9]. To further investigate ESUS-specific treatment approaches, three randomised controlled trials, NAVIGATE-ESUS, RE-SPECT ESUS, and ATTICUS, have been conducted [17–19]. Some of these trials excluded young adults with ESUS, and the generalizability of the results to young adults is unclear. Therefore, there remains a need to investigate the differences between younger and older patients with ESUS.

The findings of the present retrospective study, conducted at a single large specialised centre, showed that one in four cases of ESUS was diagnosed in younger adults. In a previously published study, in 351 patients with ESUS, 78 (22%) involved younger adults aged <50 years [20]. In a Polish cohort of patients with ESUS, patients aged <60 years comprised 32.2% of the cases of ESUS [21]. Additionally, two previously published studies have reported the prevalence of ESUS among younger adults with stroke. Ladeira et al. showed that in 100 younger Portuguese patients with ischaemic stroke, 42% had ESUS [22]. Also, a report on the Helsinki Young Stroke Registry showed that 20.9% of patients aged 15–49 years with first-ever ischaemic stroke were classified as having ESUS [23].

In the present study, younger adults with ESUS had fewer vascular risk factors, which supports the findings from previously published studies [12, 20, 22]. Reduced vascular risk factors (which include hypertension, diabetes mellitus, smoking, and dyslipidaemia) are not specific to younger patients with ESUS as they have also been reported generally in younger patients with stroke [24]. For example, among 3,944 younger European stroke patients, frequent risk factors were smoking (49%), dyslipidaemia (46%), and hypertension (36%) [25]. Perera et al. found that 21% of younger adults with ESUS had diabetes, 36% had hypertension, 28% smoked, and 28% had dyslipidemia [20]. In the present study, the severity of stroke at admission was not significantly different between the younger and older patients with ESUS, which is a finding supported by a previous study [20].

ESUS in younger adults is associated with considerable morbidity and mortality. A previously published study reported 1% mortality in younger patients during hospitalization, which was not significantly different from the mortality rate among older ESUS patients [20, 21]. In the cohort of younger patients in the present study, approximately 18% of patients with ESUS had either died or had a neurological disability when discharged from hospital. Disability at hospital discharge has not been described previously in this subgroup of patients.

This study had several limitations. First, the stroke unit admission criteria excluded elderly patients aged >80 years and patients with poor functional status before stroke (mRS score >2), dementia, or a terminal illness, which could have introduced bias resulting in an erroneously higher proportion of younger adults included in the study. Second, patients did not undergo further prolonged rhythm monitoring (insertable cardiac monitor device) follow-up after hospital discharge except in two patients who stayed in sinus rhythm. Some patients who were initially diagnosed with ESUS could have had a subsequently identified cause for their stroke. For example, 12.4% of cryptogenic stroke patients aged ≥40 years were found to have atrial fibrillation during one-year monitoring with an insertable cardiac monitor [26]. Another report showed that 16% of patients with cryptogenic stroke aged ≥55 years had atrial fibrillation after 30-day monitoring with an event recorder [27]. Three years of cardiac monitoring has been shown to identify atrial fibrillation in 33–35% of patients with ESUS [28]. Therefore, it is possible that some of the cohort of patients in the present study were misclassified as having ESUS. Third, although the mandatory diagnostic workup for ESUS does not include TEE, data suggest that TEE for patients with ESUS may alter the diagnosis and change the management in up to 16% of patients [29]. In our cohort, all patients ≤50 years had TEE compared to 15% only in the older patients. If all the patients with ESUS had undergone TEE, there is a possibility that some may have been classified as patients with cardioembolic stroke. Fourth, the small study sample size increases the likelihood of type II error (failure to detect a true difference), which may have affected the results.

## 5. Conclusion

A significant proportion of patients with ESUS are younger adults (aged ≤50 years). ESUS is a serious condition, as up to fifth of those affected younger adults may die or become neurologically disabled. The findings of this study showed that there were fewer classical risk factors for ischaemic stroke in the younger patient population when compared with older patients with ESUS.

## Figures and Tables

**Table 1:** Demographic and clinical characteristics of younger (≤50 years) versus older (>50 years) patients with embolic stroke of undetermined source (ESUS).

Characteristic	Older with ESUS (*N* = 108)	Younger with ESUS (*N* = 39)	*P*-value
Age (years). Mean ± SD	63.9 ± 8.2	42.9 ± 7.7	<0.0001

Female sex. No. (%)	39 (36.11)	11 (28.2)	0.4

Medical history. No. (%)			
Ischaemic heart disease	8 (7.4)	2 (5.1)	1.0
Arterial hypertension	76 (70.3)	17 (43.6)	0.004
Diabetes mellitus	62 (57.4)	14 (35.9)	0.03
Dyslipidaemia	31 (28.7)	5 (12.8)	0.05
Body mass index (BMI). Mean ± SD	27.8 ± 6.6	28.7 ± 5.9	0.2
History of smoking	15 (13.9)	8 (20.5)	0.3
Previous ischaemic stroke or TIA	24 (22.2)	5 (12.8)	0.2

Pre-stroke mRS (0–1). No. (%)^∗^	95 (87.9)	37 (94.9)	0.36
NIHSS score on admission. Median (IQR)^†^	5.0 (8.0)	3.0 (9.0)	0.8

Treatment with t-PA or EVT. No. (%)	5 (4.6)	4 (10.3)	0.2

ESUS, embolic stroke of undetermined source; EVT, endovascular thrombectomy; IQR, interquartile range; mRS, modified rankin scale; NIHSS, national institutes of health stroke scale; TIA, transient ischaemic attack; t-PA, tissue plasminogen activator. ^∗^mRS scores range from 0 (no neurologic deficit) to 6 (death). ^†^NIHSS scores range from 0 (normal function) to 42 (death), with higher scores indicating a greater neurological deficit.

**Table 2:** Clinical outcomes in younger (≤ 50 years) vs. older (>50 years) patients with embolic stroke of undetermined source (ESUS).

Outcome	Older with ESUS (*N* = 108)	Younger with ESUS (*N* = 39)	Effect size	*P*-value	Adjusted effect size	*P*-value
Poor outcome. No. (%)^∗^	31 (28.7)	7 (17.9)	1.84 [0.7, 4.6]	0.2	0.8 [0.1, 5.5]	0.8
NIHSS score ≤5 at discharge. No. (%)^†^	80 (74.8)	33 (84.6)	1.9 [0.7, 4.9]	0.2	0.3 [0.03, 2.4]	0.2
Median LOS (IQR) (days)	4.0 (8.5)	4.0 (7.0)	3.3 [−2.9, 9.5]	0.3	4.6 [−3.7, 12.9]	0.3

ESUS, embolic stroke of undetermined source; IQR, interquartile range; mRS, modified rankin scale; NIHSS, national institutes of health stroke scale; LOS, length of stay. ^∗^Poor outcome is defined as mRS >2 [neurological disability] or mortality at discharge. mRS scores range from 0 (no neurologic deficit) to 6 (death). ^†^NIHSS scores range from 0 (normal function) to 42 (death), with higher scores indicating a greater neurological deficit.

## Data Availability

Data is available if needed through communication with corresponding author.
